# Telehealth Movement-to-Music to Increase Physical Activity Participation Among Adolescents With Cerebral Palsy: Pilot Randomized Controlled Trial

**DOI:** 10.2196/36049

**Published:** 2022-10-28

**Authors:** Byron Lai, Laura Vogtle, Raven Young, Mary Craig, Yumi Kim, Marissa Gowey, Erin Swanson-Kimani, Drew Davis, James H Rimmer

**Affiliations:** 1 Division of Pediatric Rehabilitation Medicine Department of Pediatrics University of Alabama at Birmingham Birmingham, AL United States; 2 Department of Occupational Therapy University of Alabama at Birmingham Birmingham, AL United States; 3 Department of Physical Medicine and Rehabilitation University of Alabama at Birmingham Birmingham, AL United States; 4 Division of Pediatric Gastroenterology, Hepatology and Nutrition Department of Pediatrics University of Alabama at Birmingham Birmingham, AL United States; 5 Dean's Office University of Alabama at Birmingham Birmingham, AL United States

**Keywords:** exercise, developmental disability, cerebral palsy, telemedicine, telerehabilitation, therapy, mobile phone

## Abstract

**Background:**

Adolescents with cerebral palsy (CP) who have mobility limitations have almost no access to inexpensive and enjoyable home-based programs that can be disseminated on a large scale to help them independently manage their health through participation in leisure-time physical activity (LTPA).

**Objective:**

The primary aim of this study was to determine the preliminary efficacy of the early adoption phase of an adult Movement-to-Music (M2M) program with behavioral telecoaching for increasing LTPA and activity participation compared with a waitlist control group in adolescents with CP. The secondary aim was to explore the effects of the program on perceived levels of pain and fatigue. The tertiary aim was to qualitatively evaluate the factors that influenced adherence and develop a theory that would inform the development of a more targeted M2M telehealth program for this group.

**Methods:**

This randomized controlled trial piloted a 4-week M2M program with weekly behavioral telecoaching among 58 adolescents with CP who walked or used wheelchairs. The participants were randomized into one of 2 groups: M2M or control, which maintained their daily activities. M2M included videos that participants were asked to complete 3 times each week at home (asynchronous training). Adherence to video minutes was objectively measured using cloud-based analytics. Changes in activity and LTPA participation were measured before and after the intervention using the Children’s Assessment of Participation and Enjoyment total domain scores and active physical recreation domain scores, respectively. Perceived pain and fatigue were measured using the National Institutes of Health Neuro-QoL short forms. The changes in scores were compared between the groups using analysis of covariance. A grounded theory approach was used to analyze one-on-one interviews, coaching notes, and feedback surveys.

**Results:**

A total of 58 people were enrolled, of which 49 (84%) completed the primary outcome follow-up assessment. The mean adherence to the prescribed exercise video minutes across all 4 weeks was 68%, starting from 90% in week 1 and gradually declining to 43% in week 4. Mean adherence to coaching calls was 91%. Analysis of covariance revealed a statistically significant difference between the pre- to postchange scores for Children’s Assessment of Participation and Enjoyment Active Physical Recreation–Intensity domain scores in favor of the intervention group (*F*_1,47_=8.76; P=.005; effect size=0.17, also known as volume of LTPA). The qualitative findings highlighted 5 critical factors that influenced participants’ adherence to the program: caregiver support, video elements, suitable exercises, music, and behavioral coaching.

**Conclusions:**

This project determined that adolescents with CP responded well to an M2M telehealth program that could enhance their LTPA levels. This paper describes a theory in which adherence to a telehealth LTPA program can be optimized through functional and age-specific modifications for adolescents with CP.

**Trial Registration:**

ClinicalTrials.gov NCT04264390; https://clinicaltrials.gov/ct2/show/NCT04264390

## Introduction

### Background

Cerebral palsy (CP) is a common motor disorder in childhood [1] and “describes a group of permanent disorders of the development of movement and posture, causing activity limitations that are attributed to nonprogressive disturbances that occurred in the developing fetal or infant brain” [2,3]. Adolescence is a critical period for health professionals to engage youth with CP in regular leisure-time physical activities (LTPAs). LTPA is defined as energy expenditure from sports, conditioning, exercise, household tasks, and other daily activities [4]. Reports have indicated that young people with CP have alarmingly low rates of LTPA, far exceeding those observed among typically developing young people [5,6]. The coronavirus pandemic has further reduced participation in LTPA among youth with CP [7]. However, physically active youth with CP are more likely to live healthy active lifestyles as adults [6], which has important consequences for healthy aging with CP.

Previous studies have suggested that LTPA interventions can improve gross motor function [8], reduce morbidity risk [9], and potentially assist in the management of secondary conditions, such as pain and fatigue [10,11]. Participation in LTPA is further supported by over 3 decades of published clinical studies that reported benefits to physical fitness and well-being in children with CP [8,12-15]. Collectively, these findings demonstrate a gap between successful clinically tested programs and those that impact larger populations within the community. Thus, there is a need to identify programs that can easily be translated beyond clinical and research-supported settings to reach larger populations of people with CP.

Clinical LTPA studies targeting youth with disabilities are often not designed to be translated into the home or community and therefore *fall short* of reaching larger populations [16]. Owing to the inability to reach and include large sample sizes within randomized controlled trials (RCTs), there is little high-quality evidence (meta-analyses) that confirms the effects of LTPA interventions in CP [8]. Moreover, interventions typically prescribe programs that are supervised and delivered on-site at a fitness or clinical facility and often include only ambulatory individuals (ie, studies often exclude people who use wheelchairs) [8,17-19]. Achieving LTPA guidelines is far more difficult for people with CP who may use wheelchairs or have difficulty walking for long periods, regardless of whether general guidelines [20] or CP-specific guidelines [15] are used. In addition, there are often geographical, environmental (eg, lack of transportation), or economic (eg, cannot afford a fitness membership or one-on-one supervision by a therapist) reasons for isolation from participating in on-site LTPA programs at local facilities or clinics [21,22]. Therefore, there is an urgent need to translate successful clinical exercise studies into more widely available exercise programs that target underserved and physically inactive adolescents with CP who have mobility limitations.

Home-based exercise programs that incorporate “virtual” behavioral coaching through videoconferencing (telecoaching) are a desirable approach for people with disabilities who may not have convenient access to other means of exercise. The advantages of a telehealth approach over usual care include increased social support, quality of care, cost-effectiveness, and access to services [23]. These advantages have made telehealth an instrumental part of health services since the onset of the COVID-19 pandemic [24]. According to the supportive accountability theory [25], programs that use telehealth technology can foster strong adherence by overcoming barriers to on-site health services and promoting strong relationships with health professionals. Telehealth with behavioral coaching explains the success of 2 of the largest exercise trials for youth with disabilities [26,27], which reached sample sizes of 101 and 92 by incorporating telehealth technology to reduce the burden on both the participant and the LTPA trainer. The average sample size for the LTPA trials among youth with disabilities is 27 people [16].

To develop and implement large-scale trials for adolescents with CP, lessons can be learned from the adult literature, which has been more successful in reaching larger study samples. One ongoing telehealth trial is testing the effectiveness of a novel Movement-to-Music (M2M) video program that includes more than 400 adults with physical disabilities (NCT03024320). M2M is a 12-week program that incorporates 83 enjoyable movement routines accompanied by music to improve cardiovascular capacity, muscular strength and endurance, and range of motion. M2M combines elements of music with health-enhancing exercises, which may improve attention, communication, brain plasticity [28,29], and functional outcomes [29]. Evidence supports the efficacy of M2M in enhancing functional mobility (the Timed Up and Go and 6-Minute Walk Test) among adults with multiple sclerosis [30]. Although M2M is primarily implemented through telehealth channels, M2M was originally modified and scaled up from an on-site program that was delivered at a state-of-the-art exercise facility for people with disabilities (Lakeshore Foundation).

To date, there is no evidence-based program that can be easily disseminated by health professionals across multiple sites and settings to promote LTPA among adolescents with CP who have mobility limitations. A low-cost M2M program has the potential to address this need, but it has not been tested among children with disabilities.

### Objectives

The purpose of this study was to examine the preliminary efficacy of a youth-based adapted M2M intervention for increasing both activity and LTPA participation among adolescents with CP, compared with a 4-week waitlist control (WC) group. A secondary purpose was to explore the potential effects of the program on the levels of perceived pain and fatigue, which are 2 important secondary conditions that can likely be improved through LTPA participation. A tertiary aim was to qualitatively evaluate the critical factors that influenced adherence and to develop a theory that would inform the development of a more targeted M2M telehealth program for CP. The original M2M program included movement exercises for a larger, more general population of adults with physical disabilities.

To determine whether M2M requires specific modifications for adolescents with CP, this study aimed to address the following research question: Can the early adoption phase of an adult-based program increase physical activity, LTPA, pain, and fatigue levels among adolescents with CP? Given that the intervention included exercise videos and behavioral coaching aimed at increasing community activity, we hypothesized that participants in M2M would increase their levels of LTPA and activity after the intervention compared with WC participants.

## Methods

### Study Design and RCT Considerations

This study was a parallel-arm pilot RCT (immediate M2M intervention group vs WC group). The person entering the data was blinded to group allocation. Randomization was performed by the project statistician, who was not involved in recruitment or intervention. This trial was registered a priori as a clinical trial (NCT04264390). The trial was conducted from February 2020 to September 2021 during the COVID-19 pandemic. Owing to COVID-19 university-related delays, the length of the intervention was shortened to 4 months from the originally proposed 8 months before conducting the trial (approved by the study sponsor). In accordance with the National Institutes of Health Stages of Intervention Development, this study was a stage 1 trial. The trial focused on testing a new behavioral intervention in a pilot test and testing implementation issues that assist in the development of training materials for a larger trial. This study also included a preliminary examination of outcomes that will provide estimates of efficacy for a larger trial.

### Recruitment

Participants were recruited via telephone through medical and billing records and physician referrals from a Children’s Hospital. Recruitment was conducted between September 2020 and September 2021. The eligibility criteria included (1) a diagnosis of CP as determined by the International Classification of Disease codes from electronic medical records, (2) the ability to exercise with arms because most of the movements include arm exercises, (3) aged between 10 and 19 years (adolescent age range, as defined by the World Health Organization) [31], (4) access to a Wi-Fi internet connection at home, and (5) ability to use a device capable of viewing internet video content (television, computer tablet, laptop computer, or desktop computer). The exclusion criteria were as follows: (1) physically active (defined as >60 minutes per day of moderate to vigorous intensity exercise in a typical week) [32] and (2) complete blindness or deafness.

### Assessment Procedures

Screening, recruitment, and data collection were remotely performed. This was performed to bypass the need for transportation. Transportation to a facility deters a substantial proportion of people from participating (even for data collection) [33,34]. The assessment procedures were as follows. First, prospective participants were prescreened for age and disability type based on medical and billing records. Second, the child, adult, or caregiver was contacted to confirm their interest and eligibility. Third, prospective participants completed the assessments. They had 2 options to complete the assessments: (1) physically mailed documents that were in a large envelope containing the informed consent document and all outcome measures or questionnaires or (2) signature of a digital consent document through a secure web database REDCap (Research Electronic Data Capture; Vanderbilt University) and completion of the questionnaires through phone calls with the research staff. Children were instructed to complete the questionnaires with the assistance of a caregiver. Fourth, once consent documents were signed, participants were randomized to either the intervention or control group. After the 4-week intervention or wait period, participants in both the intervention and control groups completed a second set of questionnaires using the same option as they had done at baseline.

In addition, at the end of the 4-week intervention, all participants provided written feedback on an open-response survey asking questions related to likes, dislikes, and recommendations to improve the program. They were also provided with the option of participating in a one-on-one phone call or Zoom (Zoom Communications) semistructured interview with the principal investigator (PI). The interviews included questions related to general perceptions of the program, likes and dislikes, preferences for music and exercises, factors that affected attendance, and recommendations to improve the program. The interviews lasted no longer than 45 minutes. The participants were given US $20 to complete the interviews. The interviews were audio recorded and transcribed for analysis.

### Randomization

After baseline assessments were completed and returned, the recruiting staff contacted the project statistician to determine the participants’ allocation to a group. Participants were randomized into 1 of 2 groups (M2M or WC) with a 1:1 allocation ratio using a permuted block randomization approach. Participants were stratified based on their functional level into either a standing (Gross Motor Function Classification System [GMFCS] levels I-III) or a seated M2M program (GMFCS levels IV-V). The randomization sequence was generated a priori by the project statistician using a computer-generated random schedule in permuted blocks (SAS, version 9.4; SAS Institute). Only the statistician knew the randomization sequence (the recruitment staff, investigators, and interventionists were blinded to the randomization order), and the statistician was not involved in any other part of the intervention or recruitment process. Given the nature of the intervention and control, it was not possible to blind the participants or telecoach during the intervention.

### Intervention Procedures

#### Overview

If randomized to the M2M group, the participants were mailed the intervention equipment and instructions. Participants randomized to the WC group received the intervention after a 4-week wait period, during which they continued their normal daily activities or therapies. The participants were given US $45 for completing the questionnaires.

#### Home-Based M2M Intervention

M2M incorporated a variety of music-oriented movement routines, which were accompanied by music, to improve cardiovascular capacity, muscular strength and endurance, range of motion, and general physical function. This M2M intervention included the early adoption phase (first 4 weeks) of a larger 12-week M2M program for adults (5R01HD085186-02) [35]. M2M includes three types of video levels that are given to participants based on their GMFCS level and functional ability: (1) videos with arm and leg exercises (level 1, designated for GMFCS levels I-II), (2) videos with only arm exercises (level 2, designated for GMFCS levels III-V or wheelchair users), and (3) videos with arm and leg exercises for one side of the body (level 3, GMFCS levels I-III with weakness or movement limitation on one side of the body, ie, hemiparesis). GMFCS levels were determined during the screening call using the expanded and revised version of the GMFCS (GMFCS-E&R) [36]. Specifically, parents were verbally guided through either the GMFCS-E&R for children aged 6 to 12 years or the GMFCS-E&R for children aged 12 to 19 years [36].

The present intervention included compilations of M2M videos organized into weekly playlists. Participants were instructed to complete all videos within their playlist 3 times per week on nonconsecutive days. In week 1, the prescription generally included a total of 48 minutes of video time, which included a mixed range of motion exercise routines with guided instructions. In weeks 2 and 3, the patients were prescribed 70 minutes of video time. Week 2 included 2 mixed range of motion and muscle strengthening routines. Week 3 included 2 range of motion routines and 1 strengthening routine. In week 4, participants were prescribed 100 minutes of video time, which included 2 range of motion exercise routines, one for strength, and one for cardiovascular exercise. A new movement routine was introduced each week in a video taught by an adult instructor. The following week, the instructional video was removed and replaced with another video, which was demonstrated by an adult actor with a disability, who guided the participants through the routine. The videos have been described in detail elsewhere [35]. The music incorporated into the videos was noncopyrighted. Songs with relaxing tones and slower tempos were selected for the range of motion. Songs with faster tempos were chosen for muscle strengthening and cardiovascular exercises. If participants had difficulty using the videos prescribed, the research staff replaced them with videos that were more suitable for their functional mobility level. For example, if a participant had difficulty with the arm or leg movements from video level 1, videos in their program could be added from videos of level 2 (arm exercise only) or level 3 (exercise with one side of the body), as appropriate. The intervention doses per program level are displayed in terms of frequency, intensity, time, and type (Table 1). The participants were mailed adjustable wrist weights for the strength exercise videos. Participants were instructed to call the lead investigator immediately if they had any concerns regarding safety or had experienced any condition or injury resulting from the intervention.

This intervention used consumer-available equipment and software that could be readily replicated by other interventionists. All M2M videos were stored on a free, secure, publicly available, cloud-based video sharing service (YouTube). The YouTube account was set to “private”: only users to whom the PI gave access could watch the videos. A telecoach monitored participant adherence using the YouTube Analytics web application. YouTube Analytics automatically records watch time, type of device used to stream content, audience retention, location, date or time frame, devices, likes and dislikes, and user comments.

To access the videos on YouTube, participants were allowed to use laptop computer, desktop computer, tablet, mobile phone, or television. To increase the consistency of how the videos were watched, all participants were provided with a Google Chromecast device so that they could stream the videos to their home television, and this was the method that participants were encouraged to use. Chromecast is a low-cost device that allows users to stream content from another computer device (eg, smartphone) onto the television, and it can be purchased from most major retailers. No changes were made to the equipment or the web-based protocol throughout the intervention period.

**Table 1:** Intervention dose.

			Week 1		Week 2		Week 3		Week 4
**Level 1 (standing)**									
	Frequency	3		3		3		3	
	Intensity	Low		Low		Low to moderate		Moderate	
	Time (mm:ss)	16:20		25:05		21:08		33:17	
	Type (video, mm:ss)	ROM^a^ I (instruct)		ROM I (part, 04:55); ROM II (Instr^b^, 20:10)		ROM I (part, 04:55); ROM II (part, 05:05); STR^c^ (Instr, 11:17)		ROM I (part, 04:55); ROM II (Instr, 05:05); STR (part, 03:40); CV^d^ (Instr, 19:37)	
**Level 2 (seated)**									
	Frequency	3		3		3		3	
	Intensity	Low		Low		Low to moderate		Moderate	
	Time (mm:ss)	16:20		23:43		21:59		33:08	
	Type (video, mm:ss)	ROM I (instruct)		ROM I (part, 04:55); ROM II (Instr, 18:48)		ROM I (part, 04:55); ROM II (part, 05:05); STR (Instr, 11:58)		ROM I (part, 04:55); ROM II (part, 05:05); STR (part, 03:35); CV (Instr, 19:33)	
**Level 3 (hemiparesis)**									
	Frequency	3		3		3		3	
	Intensity	Low		Low		Low to moderate		Moderate	
	Time (mm:ss)	15:50		21:26		21:28		30:13	
	Type (video, mm:ss)	ROM I (instruct)		ROM I (part, 04:55); ROM II (Instr, 16:33)		ROM I (part, 04:55); ROM II (part, 05:05); STR (Instr, 11:27)		ROM I (part, 04:55); ROM II (part, 05:05); STR (part, 03:40); CV (Instr, 16:32)	

^a^ROM: range of motion.

^b^Instr: instructor-guided movements.

^c^STR: strength.

^d^CV: cardiovascular.

In addition to the M2M program, the intervention included behavioral coaching calls with a telecoach using the Zoom videoconference software. The participants received a total of 4 coaching calls (1 per week). Each coaching call (phone or videoconference) lasted approximately 20 minutes. Caregivers were instructed to support their children during the coaching call, considering that parental knowledge plays a significant role in determining the extent to which adolescents participate in LTPA [22,37]. Parental awareness of the benefits of LTPA, a positive attitude, and perseverance in exploring LTPA options have been identified as facilitators of LTPA behavior [37]. Parental barriers include beliefs that LTPA and sport are unimportant, concerns about the adolescent fitting in with typically developing children, and difficulties with watching the child struggle in competitive sports [37]. A caregiver was encouraged to attend coaching calls with the adolescent participant. The participant was permitted to attend the call without a caregiver in the room, but a caregiver had to be present in the home throughout the duration of the call.

The goal of the coaching calls was to enhance adherence to exercise videos and promote general LTPA and activities within the community. The calls included behavior change techniques framed within the social cognitive theory [38]. Specific strategies included confidence building, goal setting (specific, measurable, attainable, realistic, and timely goals), planned steps toward achieving the goals, instructions on proper movement techniques to increase mastery, discussions on methods to overcome barriers to participation, resolving questions related to exercise, and discussing realistic benefits that can be obtained from exercise (review recent systematic reviews of exercise for CP). The coaching call procedures are described in Multimedia Appendix 1. Coaching sessions were conducted by 2 research staff. The lead telecoach (BL) had a research background in adapted LTPA and behavioral coaching through tele-exercise for people with disabilities, in addition to 10 years of hands-on experience in exercise training for various disability groups. The assistant telecoach was a physical medicine and rehabilitation resident physician at the Children’s, who was instructed by the lead telecoach. Together, the telecoaches reviewed the procedures biweekly to maintain coaching fidelity. The telecoaches took written notes on an Excel sheet, which was archived on the university’s secure BOX server.

#### WC Participants

Participants in the WC group were instructed to maintain their usual activities for 4 weeks. After completing this period of nonintervention, the WC participants received the M2M intervention.

### Summary of Qualitative Procedures

The summary of qualitative procedures is as follows:

1. The participants who completed the intervention were asked to participate in an interview to provide feedback on how to improve the program.
2. Interested participants were scheduled for a 45-minute phone or Zoom call (participant preference).
3. The interviews were conducted by the PI, who asked 10 questions related to general perceptions of the program, likes and dislikes, preferences for music and exercises, factors that affected attendance, and recommendations to improve the program.
4. The interview was audio recorded and transcribed by a third-party company.
5. Transcriptions were double-checked for accuracy.
6. Data were coded (see the Aim 3 Analysis section).
7. A theory was created based on coding results.
8. Participants were mailed an electronic debit card for completing the interview.

### Measures or Assessments

The study outcomes were assessed using questionnaires. If the participants were unable to complete the questionnaires independently, they were permitted to assist their children.

#### Aim 1: Primary Outcome Measures

The primary outcome was pre- to postchanges in LTPA after the 4-week M2M intervention. At weeks 0 and 4, LTPA was measured using the Children’s Assessment of Participation and Enjoyment (CAPE) [39]. CAPE is used to document changes in everyday activities. CAPE provides three levels of scoring: (1) overall participation scores, (2) domain scores that reflect participation in formal and informal activities, and (3) scores that reflect participation in 5 types of activities (active physical recreation and social, skill-based, and self-improvement activities). We aimed to compare pre- and postchanges in active physical recreation activity (a measure of LTPA) and the overall total CAPE score (a measure of activity) between the study groups. Systematic reviews have reported that CAPE has adequate evidence of validity (construct and content) and reliability (internal consistency, intrarater reliability, and test-retest reliability) to support its use among adolescents with CP [40,41].

#### Aim 2: Potential Effects on Pain and Fatigue

The secondary aim of this study was to explore the potential effects of the program on perceived pain and fatigue. Perceived pain and fatigue were measured using the National Institutes of Health Neuro-QoL Pediatric Pain and Fatigue short forms [42]. The Neuro-QoL pediatric assessments are a set of health-related quality of life instruments designed to be used for children and adolescents (aged 8-17 years) with neurological conditions or disorders [43]. The psychometric properties of the Neuro-QoL measures have been tested in a variety of clinical populations but not in CP. The rationale for examining pain and fatigue was that treatments for managing these conditions have been identified as a top research priority by CP stakeholders [44]. The Neuro-QoL Pain short form includes 8 questions pertaining to perceived pain level in the past 7 days. Questions probe perceived pain intensity, frequency, pain location, pain presence, and how pain affects lifestyle activities. The fatigue short form includes 8 questions pertaining to fatigue presence and how fatigue levels affected lifestyle activities and mood in the past 7 days. A higher score indicates a lower level of pain and fatigue.

### Aim 3: Theory Generation to Inform Future Telehealth LTPA Programs

The grounded theory framework by Charmaz [45] underpins the qualitative component of this study. This method posits that theories are constructed “through our past and present involvements and interactions with people, perspectives, and research practices.” Thus, all study components were guided by the following philosophical assumptions: a critical realism ontological perspective (ie, there is a singular reality that can be understood through subjective perceptions or experiences of events that are linked with reality) [46] and an interpretivism epistemological perspective (ie, knowledge is socially constructed by both the participant and researcher) [47]. In other words, participants recalled reality when thinking about the questions in relation to their past experiences, and this reality was influenced by the interaction of the interviewer, caregiver, and participant. The theory was informed by 3 sets of data: the one-on-one postintervention interviews, the postprogram feedback survey, and notes taken by the 2 telecoaches after each behavioral coaching call.

### Ethical Considerations

The study protocol was approved by the University Institutional Review Board for Human Use at the University of Alabama at Birmingham (300004608). Approval was obtained from the university institutional review board before starting the study. Written informed consent was obtained from all participants or their caregivers before enrollment.

### Statistical Analysis

#### Overview

Statistical significance was evaluated at a family-wise error rate of 0.05. Quality control included data double-checking as well as descriptive and graphical approaches to summarize the baseline characteristics of all key variables, followed by independent 2-tailed *t* tests and chi-square statistics to assess baseline group differences. All data were double-checked for their accuracy.

#### Power

A power estimate calculation determined that a sample size of 54 was needed to detect a group difference, given the following variables: significance level of 0.05, Cohen *d* of 0.75, and power of 80%. The calculation was based on data obtained from an RCT of exercise for children and adolescents with CP that used the same measure for LTPA participation (CAPE) [48]. Although the duration of the intervention was longer than that in this study, the team decided that the intervention and measures were most relevant compared with other randomized trials [19].

#### Primary Analysis

We examined the effects of the intervention versus control condition on pre- to postchanges in LTPA (CAPE total scores, domain scores, and CAPE Active Physical Recreation [CAPE-APR] domain scores) using analysis of covariance (ANCOVA) based on the univariate *F*-statistic (conditions: intervention and control, change score, and baseline variable data as the covariate). ANCOVA has been found to be valuable as an effective and nonbiased method of analyzing pre- to postchange scores [49], and it has the smallest variance, highest power, and nominal 95% CI coverage compared with ANOVA and linear mixed models [49,50]. This method was also used to compare group differences between pre- and postintervention changes in secondary outcomes. If ANCOVA assumptions were not met, 2-tailed *t* tests were conducted.

#### Tertiary Qualitative Analysis

Audio transcriptions, participant feedback surveys, and telecoach notes were considered qualitative data that were analyzed using a grounded theory approach. The coding process included 3 phases: generation of (1) initial codes (ie, phrases that represent lines of text), (2) focused codes (ie, phrases that represent one or more initial codes), and (3) conceptual categories (ie, higher-order phrases that represent one or more focused codes) [45]. Conceptual categories and codes were arranged into a conceptual map (substantive theory) to address the study objectives. This process has been described in detail elsewhere [51]. The 2 analysts were the telecoaches. The lead analyst was a qualitative researcher with experience conducting more than 350 interviews for people with disabilities. The second analyst, a medical resident, was trained by the analyst.

#### Data Exclusion

Data from participants who did not complete the postintervention assessments were excluded from the analyses.

## Results

### Quantitative Results

#### Overview

The participant characteristics are presented in Table 2. Detailed recruitment and enrollment information is displayed in a CONSORT (Consolidated Standards of Reporting Trials) diagram (Multimedia Appendix 2). Of the 521 people contacted, 82 (15.7%) were assessed for eligibility, 58 (11.1%) were enrolled, 51 (9.8%) completed the intervention, and 49 (9.4%) completed the intervention and assessments and were included in the analyses. A total of 4 participants did not want to complete the pain and fatigue measures because they felt overburdened by the assessments and the paperwork. At baseline, there were no statistically significant differences between the groups in terms of study outcomes or characteristics (all P>.05).

The mean adherence to the prescribed exercise video minutes across all 4 weeks of the program was 68% (all 49 people, immediate start and control). The mean adherence to the videos was 90% (44/49 minutes) in week 1, 83% (56/68 minutes) in week 2, and 69% (45/65 minutes) and 43% (40/95 minutes) in weeks 3 and week 4, respectively. Adherence to coaching calls was 98% in week 1, 90% in week 2, and 90% and 86% in weeks 3 and 4, respectively. No adverse events (eg, accidents, injuries, or conditions related to the intervention) were reported by the participants.

**Table 2:** Participant characteristics (n=49).

Characteristics			Values
Age (years), mean (SD)			14 (3)
**Sex, n (%)**			
	Male	25 (51)	
	Female	24 (49)	
**Ethnicity, n (%)**			
	White	30 (61)	
	Black	17 (35)	
	Asian	2 (4)	
	Hispanic or Latino	1 (2)	
**Gross Motor Function Classification System** **level I-V, n (%)**			
	I	7 (14)	
	II	16 (33)	
	III	6 (12)	
	VI	11 (22)	
	V	9 (18)	

#### Aim 1 and 2 Outcomes

The pre- to postchanges in outcomes between the groups are displayed in Table 3 for outcome variables. ANCOVA revealed statistically significant between-group differences in pre- to postchange scores for CAPE-Intensity (*F*_1,47_=5.63; P=.02; effect size=0.11; also known as volume of *activity*) and CAPE-APR–Intensity (*F*_1,47_=8.76; P=.005; effect size=0.17; also known as volume of *LTPA*). The estimated marginal means revealed that these were small changes in favor of the intervention group. Interpretation of CIs revealed that changes in activity intensity could be classified as minimal to no changes because 0 was included within the CI [52]. There were no statistically significant group differences in overall CAPE score or CAPE-APR–Diversity, –With Whom, -Where, or -Enjoyment scores (all P>.05). There were no statistically significant group differences in the pain or fatigue raw scores (all P>.05).

**Table 3:** Pre- to postchanges in outcomes by intervention group and time.

Variable	M2M^a^ change in score, mean (SD; CI)	M2M (n=23), n (%)	WC^b^ change in score, mean (SD; CI)	WC (n=26), n (%)	*F* value (*df*)	P value
CAPE^c^-Diversity	−0.43 (3.59; −2.0 to 1.47)	23 (47)	0.19 (4.72; −1.59 to 1.67)	26	0.065 (1,47)	.80
CAPE-Intensity	0.10 (0.31^d^; −0.06 to 0.26)	23 (47)	−0.16 (0.43^d^; −0.31 to −0.01)	26	5.75 (1,47)	.02^d^
CAPE–With Whom	0.02 (0.41; −0.18 to 0.23)	23 (47)	−0.05 (0.66; −0.24 to 0.14)	26	0.29 (1,47)	.59
CAPE-Where	−0.03 (0.52; −0.20 to 0.19)	23 (47)	0.11 (0.42; −0.09 to 0.27)	26	0.52 (1,47)	.47
CAPE-Enjoyment	−0.24 (0.99; −0.54 to 0.06)	23 (47)	−0.05 (0.44; −0.33 to 0.23)	26	0.93 (1,47)	.34
CAPE-APR^e^–Diversity	0.35 (1.56; −0.32 to 1.02)	23 (47)	−0.15 (1.05; −0.57 to 0.27)	26	1.79 (1,47)	.18
CAPE-APR–Intensity	0.35 (0.62^d^; 0.11 to 0.58)	23 (47)	−0.18 (0.54^d^; −0.4 to 0.041)	26	5.76 (1,47)	.02^d^
CAPE-APR–With Whom	0.03 (1.15; −0.69 to 0.6)	23 (47)	0.48 (1.74; −0.11 to 1.07)	26	1.45 (1,47)	.23
CAPE-APR–Where	−0.03 (1.23; −0.66 to 0.64)	23 (47)	0.54 (1.75; −0.1 to 1.13)	26	1.37 (1,47)	.24
CAPE-APR–Enjoyment	−0.10 (1.43; −0.66 to 0.44)	23 (47)	0.02 (1.18; −0.49 to 0.53)	26	0.12 (1,47)	.72
Pain	2.48 (5.86; 0.3 to 6.43)	21 (46)	−0.44 (7.5; −0.64 to 5.32)	25 (54)	1.75 (1,47)	.19
Fatigue	−0.09 (7.9; −2.55 to 2.79)	21 (46)	0.03 (4.68; −0.48 to 0.25)	25 (54)	0.22 (1,47)	.88

^a^M2M: Movement-to-Music.

^b^WC: waitlist control.

^c^CAPE: Children’s Assessment of Participation and Enjoyment.

^d^Significant difference at α level .05.

^e^CAPE-APR: Children’s Assessment of Participation and Enjoyment Active Physical Recreation.

### Qualitative Results

#### Overview

A total of 28 people who completed the intervention also completed the interviews, and we chose to halt the interviews at this sample size because the conceptual categories appeared saturated (ie, the data analysts felt confident that the conceptual categories were adequately represented and explored) [45,53]. The results are organized into 2 sections. The first section briefly describes the conceptual categories and codes that represent participants’ preferences for an ideal home-based telehealth LTPA program. There were 6 resultant conceptual categories that are listed in Table 4 and described in further sections. The second section elaborates on the relationships between the conceptual categories and describes a theoretical framework that can be used by interventionists and health professionals aiming to develop or implement a telehealth LTPA program for adolescents with CP.

**Table 4:** Qualitative themes.

Conceptual categories	Focused code
Enjoyment is influenced by music, suitability of the exercises, and caregiver support	Enjoyment increases attentional focus on the videos Music was the core influencer of video enjoyment and the decision to join the program Caregivers participating with their child led to noticeable improvements in a child’s mood and attention to the videos Physical assistance from a caregiver created feelings of happiness and enhanced caregiver-child bonds
Caregiver support increases the usability and suitability of the exercises	Caregivers are instrumental in addressing physical, cognitive, and motivational demands and can modify exercises Caregivers are the primary method for exercise adaptations in a home-based environment Caregiver scheduling was a direct influencer of attendance
Music is instrumental for joining and sustaining an exercise regime	Participants desired music that matched the theme of the movements (tempo and movements that are performed rhythmically to the music) Personalized music can increase enjoyment and adherence. Music need not be genre specific but should be upbeat Music was identified as critical component of the children’s lifestyles and was incorporated in most enjoyable activities they performed.
Video elements influence usability and suitability of the exercises	Most participants appreciated and preferred short and asynchronous (video-based) home exercise program because of the convenience of fitting it into their busy schedules Exercises should be adapted to include slow verbal instructions, different body positions (eg, bed or chair), and repetitive movements. These adaptations will help accommodate cognitive needs in the presence of intellectual disability (which influences the suitability of the exercises) or physical needs in the presence of impaired physical function. Doing so provides a sense of accomplishment and happiness while avoiding frustration and loss of attention Videos should include child-appropriate themes (eg, superhero, sci-fi, western, pop dance, cartoons, and sports) and relatable actors (eg, children of similar ability and mobility as the participant or cartoon characters) Exercises should be perceived as beneficial for range of motion or mobility
Behavioral coaching influences caregiver support, usability, and adherence	Caregivers strongly noted that meeting with a coach created a sense of accountability that was a key factor that affected adherence to the program Knowing that the program was offered by a trusted provider and created by a recognized community fitness facility for people with disabilities enhanced attendance and was a key factor in their decision to join the program Developing a social bond with the participant and caregiver increased accountability The coach resolved technical issues throughout the first 2 weeks of the program that had negatively impacted adherence Participants and caregivers relied on the knowledge of the coach for guidance on the prescription (instructions at baseline were not sufficient)

#### Enjoyment Is Influenced by Music, Suitable Exercises, and Caregiver Support

Adherence to the program was highly dependent on the children’s enjoyment of the program. Enjoyment with the program increased the child’s attentional focus on the movements and instructions within each video. High levels of enjoyment led to laughter and smiles when performing the movements, whereas low levels of enjoyment led to low levels of video minutes performed and noninterest in participation or even dropout (2 cases). The factors that contributed to enjoyment included elements of music, suitable exercises, and caregiver support.

#### Caregiver Support Increases Adherence and Suitability of the Exercises

Caregivers were a key factor in adherence and could perform supplemental adaptations that were needed for the child (in addition to those shown in the videos). Among participants with GMFCS levels IV to V, caregivers were instructed to modify the program for their child by physically assisting them through the movements. In these cases, caregivers reported that they noticed that the children became happy when the caregiver physically put their hands on them to perform fun and enjoyable movements, as opposed to care activities. Moreover, caregivers often managed the child’s exercise schedule and coaching calls, which were difficult because participants often had busy daily routines with school and care activities. In some cases, caregivers performed the videos with the child and used verbal cues and positive reinforcement to increase the child’s adherence.

#### Music Is Instrumental for Joining and Sustaining an Exercise Regime

Music was identified as a core component of both the child’s and caregiver’s interest in joining and participating in the program. Participants reported a love for music that was incorporated into their most enjoyable activities they did at home. Some participants loved dancing to music at home or listening to music to ease their anxiety and improve their mood. Participants expressed a variety of musical interests and noted that music aligned with their interests could enhance adherence. Some enjoyed the noncopyrighted music within the intervention, while others preferred music that aligned with their interests. However, for exercise, they reported that the music does not need to be limited to a specific genre, instead the music should coincide with the rhythm of the exercise movements (eg, an “upbeat” tempo for cardiovascular exercise). Participants strongly noted that an exercise program without a substantial musical component would not be appealing to them to participate in or perform for a prolonged period.

#### Video Elements Influence Usability and Suitability of the Exercises

Participants and caregivers reported several video elements that affected the child’s adherence. First, participants preferred short and asynchronous videos to real-time instructions (either in-person or videoconference instruction). Several participants suggested a duration <20 minutes. This was because caregivers and children had busy schedules with school, care activities, and frequent medical or therapeutic appointments or concerns. The ability to pause the videos and work at the child’s own pace was a strong advantage of asynchronous training. Second, videos were recommended to contain repetitive exercises and visual-verbal guidance to accommodate various learning and physical needs. Specifically, the fourth week of the cardiovascular exercise videos had too many transitions between exercise movements. In addition, the movements were too fast. These 2 factors were the reasons for the low video adherence at week 4. Participants also desired videos of shorter duration to accommodate short attention spans, which was the rationale for declining adherence in weeks 3 and 4. Notably, caregivers desired more program adaptations for people with GMFCS levels IV to V. In these cases, caregivers and participants desired a caregiver-child–assisted M2M program. Such a program could display a caregiver physically assisting the child throughout the movements and more appropriate exercise positions to avoid the need for transfer (eg, exercises performed in a bed, power wheelchair, or floor). For children with GMFCS levels I to III, exercises often appeared suitable and when performed successfully, provided participants with a sense of happiness and accomplishment that increased enjoyment. “Honestly, he reacted better to the videos than I anticipated...He uses a wheelchair and enjoyed them. Doing something he knows he can do. He looks forward to it” [caregiver of participant 22]. Third, participants and caregivers reported that age-appropriate themes and relatable actors could increase their interest. Actors could have a similar functional ability or be superheroes or animated characters from a show or movie. Participants desired videos based on age-appropriate themes (eg, superhero themes, pop dance themes, or sports). Fourth, exercises that were perceived as beneficial to physical function appealed to both the participants and caregivers. As stated by participant 47, “I really liked doing the exercises because I had arm surgery, and I feel like the exercises helped my arms and made me feel better.”

#### Behavioral Coaching Increases Adherence Through Accountability and Support

Both caregivers and participants reported that the program would not have adhered well without behavioral coaching support. Coaching helped in identifying movement adaptations, understanding the importance of exercise for CP, and reviewing the program structure or instructions. Most importantly, the coaching provided participants and caregivers with a sense of accountability. They felt that they had made a commitment and built a social bond with a trusted health professional, which made them want to try their best to attend the sessions. They further appreciated that a health professional was aiming to improve their child’s health outside the medical and school setting, which they felt was underserved. In addition, the coach was relied on to resolve streaming issues and provide guidance for completing the exercise prescription.

#### Theory for Increasing Adherence to Telehealth LTPA Programs

**Figure 1:**
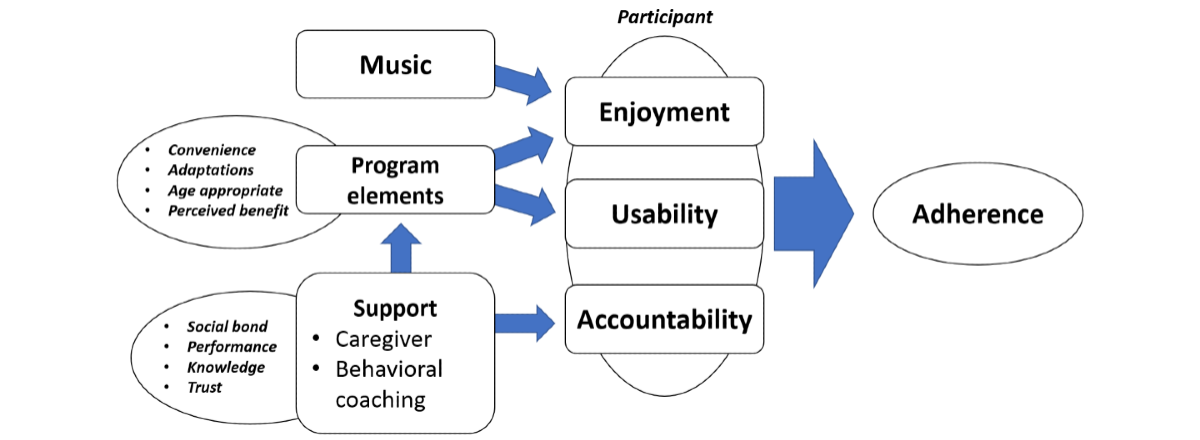
Theory for maximizing adherence in telehealth physical activity among adolescents with cerebral palsy.

This section describes a theory that can be used by interventionists and health professionals (Figure 1) for the development and implementation of a telehealth program for adolescents with CP. The theory displays a framework that depicts linkages between conceptual categories that may lead to increased adherence to a telehealth LTPA program.

The theory posits that 3 elements influence the adherence of adolescents with CP to a telehealth LTPA program: support, video elements, and music. A successful telehealth program aimed at promoting self- or family-regulated home exercise behavior should address all 3 elements. Adherence is influenced by enjoyment, usability (ie, successful video performance), and accountability. Music is a strong factor of enjoyment and should be considered mandatory in any home-based exercise program that aims to promote sustainable exercise behavior. Enjoyment is further influenced by program elements (eg, children are happy to perform movements that match their functional ability and include age-appropriate themes and actors) and support from both the caregiver and behavioral coach.

The program elements influence adherence and usability. If an exercise is not adapted for the individual, they will not be able to perform the movement successfully and as a result, rapidly lose interest. Moreover, despite suitable exercises, if the video instructions were not presented appropriately, the exercise would not be suitable for the participants’ needs. Thus, video elements are indirectly linked to usability.

Caregiver influence was critical to participant adherence and often outweighed other factors. For example, a highly involved caregiver could increase adherence to less enjoyable music and unsuitable exercises or video elements. Behavioral coaching was critical for maintaining the caregivers and participants involved by providing a sense of accountability through performance monitoring, knowledge of benefits, and a bond with a trusted health professional or organization. This theory was underpinned by the supportive accountability theory [24]. In addition, coaching helped resolve programmatic and technological issues and helped caregivers engage in program adaptations. Thus, behavioral coaching influences adherence through accountability and indirectly through caregiver support, along with an indirect influence on adherence through program elements.

Conceptually, all 3 constructs of the theory, namely music, program elements, and support, should be addressed to enhance the likelihood that adolescents with CP adhere strongly to a telehealth LTPA trial.

## Discussion

### Principal Findings

Although there are efficacious on-site LTPA interventions for adolescents with CP, the effectiveness and dissemination of these interventions are low because of accessibility and expense. Recent systematic reviews have demonstrated that LTPA trials are only effective at increasing LTPA participation among ambulatory people with CP and typically include small sample sizes [16,19]. This project piloted a low-cost behavioral coaching M2M program among adolescents with CP to determine whether the telehealth program could potentially improve activity, LTPA, pain, and fatigue and whether modifications were necessary.

Quantitative findings demonstrated that the M2M program resulted in a small increase (effect size=0.17) in the volume of LTPA performed by the individual in the home setting compared with WC. This was demonstrated by a statistically significant improvement in CAPE-APR–Intensity but no improvement or difference in CAPE-APR–Diversity (types of activities), –With Who (exercising with others), –Where (exercise setting), and –Enjoyment scores. A statistically significant increase in nonsupervised LTPA behavior is meaningful, given that very few RCTs have successfully promoted the adoption of independent exercise behavior within the community [16,18,19], particularly among people with CP who have higher mobility disability. A meta-analysis by Reedman et al [18] demonstrated that behavioral interventions are efficacious in improving “habitual physical activity,” measured via steps per day, but interventions have not been effective in improving LTPA. In other words, interventions with behavioral coaching can improve step count among people who are ambulatory. However, further research is warranted into methods of promoting LTPA within the community among people who are nonambulatory. Although there was a statistically significant improvement in general activity (CAPE-Intensity), the difference was not strong enough to be termed an effect.

People with CP who use wheelchairs have largely been excluded from past LTPA interventions. A scoping review of LTPA studies reported that of the 48 RCTs including 1513 people with CP, only 16 people were reportedly included with a GMFCS level V, and 64 people with a GMFCS level IV were included [19]. In a recent report by Gross et al [44] and the Cerebral Palsy Research Network, the development and evaluation of effective methods for increasing LTPA among people who are less ambulatory have been ranked as a top research priority by a large cohort of CP stakeholders.

A strength of this study was that there was a strong representation of GMFCS levels IV and V. People with these levels were able to be included by adapting the exercise movements. Telecoaches guided caregivers to physically assist the participants with their exercises. This was done by the caregiver moving the participant’s arm while following the video. This often required several pauses by the caregiver to allow adequate time to learn a movement or adjust the participant’s position. A valuable and unexpected outcome was that the caregiver physically assisting the child created sensations of happiness and joy, which was caused by the child associating physical touch with something fun and playful, as opposed to daily care. Nevertheless, caregivers and children expressed a strong desire for caregiver-assisted videos and general exercise knowledge for their children, which was directly applicable to their scenarios, and this should be a research priority.

Regarding program adherence, we deemed an overall adherence rate of 69% to be acceptable. This determination was based on evidence that adherence rates between 50% and 69% to an on-site 12-week M2M program resulted in improved functional mobility among adults with physical disabilities [30,54]. Nevertheless, adherence was strong during the first 2 weeks of the program and declined gradually throughout weeks 3 and 4. Qualitative study findings demonstrated that adherence declines were due to the increasing duration of the videos as well as the too fast and complex cardiovascular routine provided in week 4. On the basis of the qualitative study findings, we recommend that asynchronous video programs should be brief in duration and include repetitive cardiovascular exercise routines with age-appropriate themes.

Although we were slightly shy of our target recruitment goal, another strength of this study was that a respectable sample size was obtained within a 1-year time frame. The achieved sample size was nearly twice the mean sample size of LTPA trials for children with disabilities [16]. Nevertheless, the findings demonstrated that the intervention had no effect on the level of perceived pain or fatigue. There are several possibilities for this. First, the dose was likely insufficient to improve the pain. Only a handful of studies have suggested potentially beneficial effects of exercise on perceived pain and fatigue among people with CP [11,19,55]. The 2 studies that reported benefits to pain and fatigue included resistance training programs that were higher in intensity than in the first 4 weeks of M2M. A second explanation was that the baseline values for pain were low, leaving little room for improvement. The mean baseline scores were 1.6 and 2.4 for pain and fatigue, respectively. These scores indicated “never/almost never” feeling pain and “a little bit/some of the time” feeling fatigue. Nevertheless, further efforts are needed to examine the effects of exercise on pain and fatigue in people with CP [19].

Several novel features were embedded in the project. First, the telehealth intervention enhanced participant adherence by providing remote support from a disability exercise professional. Second, the telehealth project overcame common logistical barriers (eg, transportation, time, and program costs) that prevented adolescents with CP from participating. Third, the intervention procedures were specifically designed to be implemented with minimal resources (eg, low-cost prerecorded videos, no transportation required, and minimal equipment) and burden on research staff to facilitate replication of the project in a larger future trial.

### Recommendations for Future Telehealth LTPA Trials

Qualitative findings identified critical factors that contributed to participants’ adherence, and these findings were packaged into a theory that can maximize adherence in future telehealth LTPA trials. The 5 constructs of the theory, namely music, caregiver support, video elements, suitable exercises, and coaching, provide a general guide for replicating the study procedures in a future telehealth trial that can foster enjoyment, usability, and adherence. Specific intervention content (eg, choice of music and specific exercises) should be tailored to the needs and preferences of the specific target population. For example, the participants in this study were located within the Southeast United States, and their preferences will certainly not be generalizable to all adolescents with CP. This information can be obtained from a quick survey or usability study. In further sections, we elaborate on how these factors affect adherence to our intervention and list recommendations for future LTPA telehealth trials.

Attendance at the program was strong during the first 2 weeks of M2M. Participants had passion for music and felt that the program came from a trusted source (the hospital and adapted fitness facility). These factors also heavily influenced their decisions to participate in the program. However, adherence to week 3 declined as the movements became more numerous and complex, and the videos became longer. A large drop in attendance was observed during week 4, when the first cardiovascular exercise video was introduced, which had many movements that transitioned too quickly for many participants to follow. Caregiver support was often sufficient to counterbalance these inadequacies. Several lessons can be learned to further increase participant adherence in future trials:

1. Exercises and their instructions should be repetitive with slow transitions between different movements.
2. Exercises should always be visually guided, in addition to verbal instruction.
3. Music should be integrated into all components of the program (even the instructions).
4. Music preferences vary, and thus, the choice of music should emphasize the upbeat tempos that are specific to the target group.
5. Videos should include actors with disabilities that match the mobility and functional abilities of the participants.
6. Telecoaches should have behavior change techniques readily available for both the caregiver and adolescent.
7. Videos should be presented with adolescent-appropriate themes.

### Limitations

This pilot study has several limitations. First, the study did not include any objective measures of health or physical activity, which should be the focus of a future trial testing a CP-specific M2M program. Second, the study was implemented during the COVID-19 pandemic, which could have influenced the response rates and changes in outcomes. For example, participation in community activities or physical activities may have been resistant to change, considering that the state was in lockdown, and many community activities were closed or had occupancy limits. By contrast, this could have inflated our response rates, as participants may have been more interested in a home exercise program during this period. Third, our sample was short of the target by 5 people. Fourth, this project initially aimed to test an 8-week M2M program. Owing to the COVID-19 restrictions, we experienced a 6-month delay in university-related operations, which required us to shorten the intervention to complete the study within a 1.5-year period. There is a need to test for longer intervention and follow-up periods to assess sustainability. Fifth, with only 2 study groups (intervention and control), we were unable to discern how physical activity levels would differ with and without behavioral coaching (ie, how behavioral coaching affects adherence), which should be tested in a future trial with 3 study groups (intervention with behavioral coaching, intervention alone, and control). Finally, the intervention required participants to have access to wireless internet at home, which may affect the generalizability of the study findings. No participants were excluded because they did not have access to wireless internet at home.

### Conclusions

This project established the preliminary efficacy of a scalable telehealth M2M program for increasing LTPA behavior among adolescents with CP. Overall, participants responded well to the program, which improved their LTPA levels at home. Adherence to the program was strong during the first few weeks but declined as the exercises became less suited to the participants’ needs. These findings demonstrated that M2M may require additional CP-specific modifications before implementation in a larger multisite trial. Ideally, the final product will result in an exercise program that can be implemented across a variety of settings to reach and include an underserved population of adolescents with mobility limitations.

## Appendix

Multimedia Appendix 1 Coaching operation procedures.

Multimedia Appendix 2 CONSORT (Consolidated Standards of Reporting Trials) diagram.

Multimedia Appendix 3 CONSORT-eHEALTH checklist (V 1.6.1).

## Abbreviations

ANCOVA: analysis of covariance
CAPE: Children’s Assessment of Participation and Enjoyment
CAPE-APR: Children’s Assessment of Participation and Enjoyment Active Physical Recreation
CONSORT: Consolidated Standards of Reporting Trials
CP: cerebral palsy
GMFCS: Gross Motor Function Classification System
GMFCS-E&R: expanded and revised version of the Gross Motor Function Classification System
LTPA: leisure-time physical activity
M2M: Movement-to-Music
PI: principal investigator
RCT: randomized controlled trial
REDCap: Research Electronic Data Capture
WC: waitlist control
