# Supplementary material for: Telehealth Movement-to-Music to Increase Physical Activity Participation Among Adolescents With Cerebral Palsy: Pilot Randomized Controlled Trial
Source: JMIR Form Res. 2022 Oct 28;6(10):e36049. doi: 10.2196/36049 (PMC9652735; doi:10.2196/36049)
Supplement: Multimedia Appendix 1 [file formative_v6i10e36049_app1.docx]

# Behavior Coaching Procedures

The coaching calls will last no longer than 30 minutes and taper off in frequency over the 8-weeks (1 call at Week 1; 1 call at Week 2; 1 call at week 4; one call at week 6). The goal of the coaching calls will be to enhance adherence to the exercise videos and promote general physical activity within the community. The calls will include behavior change strategies framed within the Social Cognitive Theory (Bandura, 2004). Specific strategies will include, goal setting (specific, measurable, attainable, realistic, and timely [SMART] goals), planned steps towards achieving the goals, instructions on proper movement techniques to increase mastery, monitoring physical activity duration in a journal, discussions on methods to overcome barriers to participation, resolve questions related to exercise, and discuss realistic benefits that can be obtained from exercise (review recent systematic reviews of exercise for CP).

***The following 4 pages contain an outline/checklist for each of the 4 coaching calls***

## 4 Coaching Calls per Participant (~20 mins each):

**Coaching Call #1: Start of week 1**

Call Outline: Establishing a relationship, program overview and guidance, and understanding exercise benefits.

1. Introductions, establishing a social bond, and introduction to the program (emphasize trustworthiness, expertise, and kindness)
   1. Introduce background (where you are from and, expertise: particularly, if it relates to exercise and disability)
   2. Introduce fun fact about self
   3. Ask adult/child to introduce themselves and give a fun fact
   4. Ask adult/child what they like to do for fun
   5. Briefly mention what trainer likes to do for fun
2. Note that the purpose of today’s call is to guide them through how to do the program
3. Ask if there were any issues with intervention equipment setup or technology
4. Assist participant and parent with equipment/technology issues if necessary
5. Introduce the program and physical activity (legitimacy)
   1. Introduce the M2M program and how it originated
   2. Emphasize that "the goal of the program to increase their health and well-being through participation in physical activity”
6. Briefly summarize the benefits of the most recent systematic reviews and meta-analyses from physical activity participation
7. Explain that the goal of the program is for them to perform all the prescribed videos, and, when they feel comfortable with the videos, also do more outside physical

activity in their community if possible.

1. Ask if they have any questions about physical activity or exercise?
2. Inform them that the minimum recommendation for this program is to: watch, follow-along with, and complete all the videos given to them 3 times per week.
3. Ask if they have any questions
4. Explain the importance of setting goals and that we will go over goal-setting in more detail on the next call.
5. Describe safety tips for exercising with the videos
   1. Instruct them to look out for dizziness, shortness of breath, chest pain or any pain
   2. Instruct them to exercise in a safe space (no obstacles or hazards nearby).
   3. Instruct them that they can do the exercise in the sitting position and not have to stand if they do not want to
6. Schedule/confirm next coaching call and end chat

**Coaching Call #2: Start of week 2**

Call Outline: Trouble shooting and creating SMART goals

1. Introduction (Say hello; it’s good to see them; ask how they are; ask how their last week was, anything exciting or eventful?)
2. Give verbal praise if you saw they watched the video (from analytics).
3. Ask: How did you feel about exercising with the videos last week?
4. Ask if there are any issues with technology or equipment and resolve them
5. Ask if the participant or parent has any questions for me about exercise, physical activity, or the videos
6. Ask if the participant or parent has any questions for me about exercise, physical activity, or the videos
7. Introduce SMART goals
8. Ask participant if there are any things they would like to participate in in the community (e.g., park, event, concert, club, sport competition, etc.)? Help them identify something if possible.
9. Help them create a SHORT-TERM goal (one-week goal)
10. Go through the SMART components and ensure it is SMART
11. Inform them that they now have two goals, stick with their goal of the exercise videos and the new community participation goal
12. Schedule/confirm next coaching call and end chat

**Coaching Call #3: Start of week 3**

Call Outline: Trouble shooting, update on goals, and verbal praise.

1. Introduction (Say hello; it’s good to see them; ask how they are; ask how their last week was, anything exciting or eventful?)
2. Ask: How did you feel about exercising with the videos last week?
3. Ask if there are any issues with technology or equipment and resolve them
4. Ask if participant was able to achieve the community goal? (support them verbally just as #14)
5. Congratulate participant on making it halfway through the program, and inform them that they should feel confident and good about themselves that they made it this far. Encourage them to keep it up and continue to achieve their goals, because there is only one more coaching call left!
6. Provide verbal praise for any major milestones that the person has accomplished in the first 4 weeks
7. Ask if the participant or parent has any questions about exercise, physical activity, or the videos

**Coaching Call #4: Start of week 4**

Call Outline: Goals update, final progress report, tips to maintain physical activity behavior

1. Introduction (Say hello; it’s good to see them; ask how they are; ask how their last week was, anything exciting or eventful?)
2. Ask: How did you feel about exercising with the videos last week?
3. Ask if there are any issues with technology or equipment and resolve them
4. Ask if participant was able to achieve the community goal?
5. Congratulate participant on making it this far in the program. Encourage them to continue with their behavior because this will be the last time that we talk until the program ends.
6. Discuss tips and strategies to maintain behavior over the long-term. Explain what a relapse is and that it is not unusual to relapse into sedentary behavior. Explain physical deconditioning and disability associated energy expenditure and how physical activity makes it easier to do your daily activities.
7. Ask if the participant or parent has any questions for me about exercise, physical activity, the videos, or the program.
8. Inform participant of when surveys will be sent and to fill them out in entirety
