# Supplementary figures and images for: Telehealth Movement-to-Music to Increase Physical Activity Participation Among Adolescents With Cerebral Palsy: Pilot Randomized Controlled Trial
Source: JMIR Form Res. 2022 Oct 28;6(10):e36049. doi: 10.2196/36049 (PMC9652735; doi:10.2196/36049)

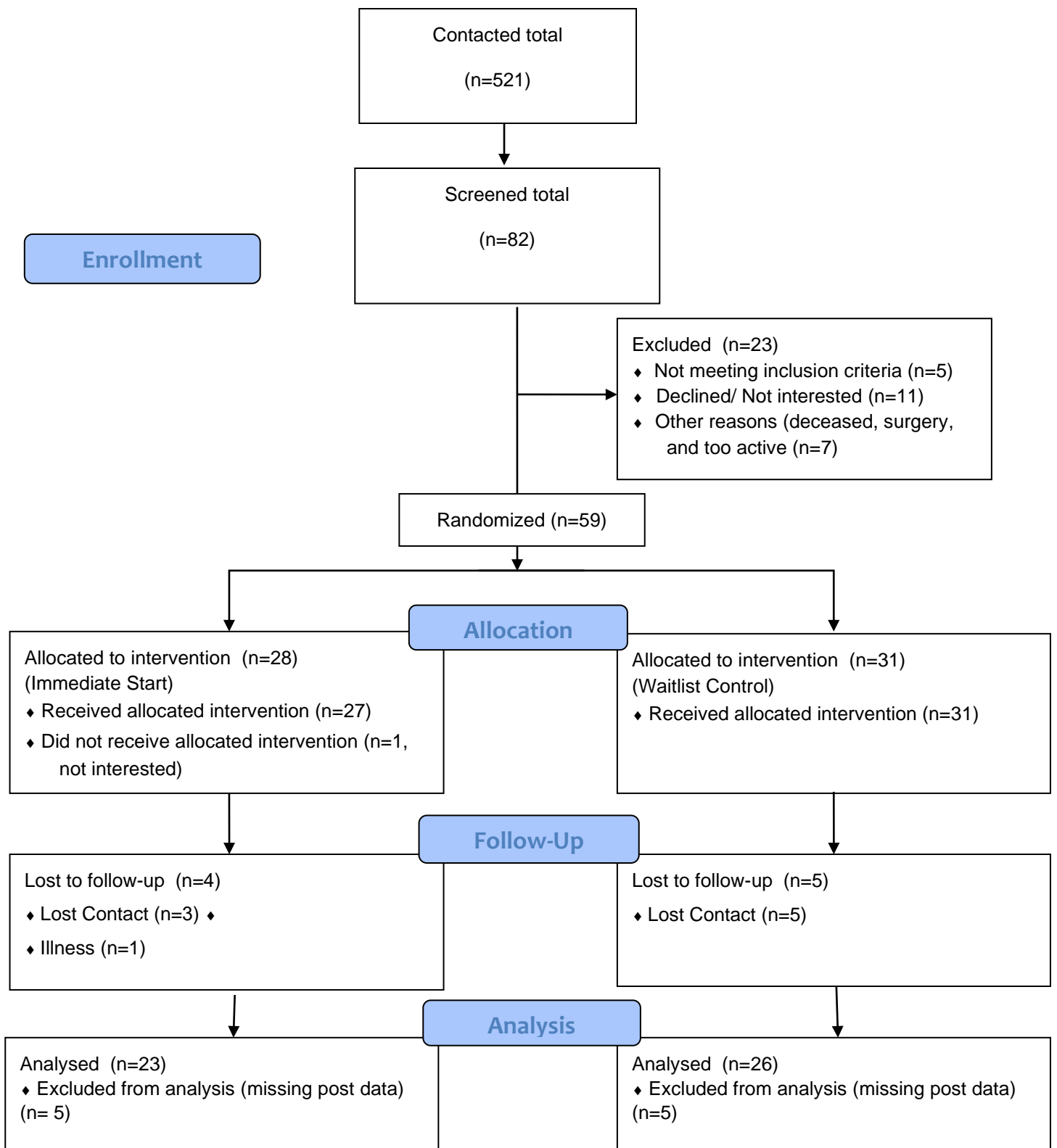

Supplement: Multimedia Appendix 2 [file formative_v6i10e36049_app2.pdf]
